# Blood Monitoring Practices in Older Adult Psychiatric Inpatients: A Retrospective Audit

**DOI:** 10.1192/bjo.2026.11726

**Published:** 2026-06-30

**Authors:** Rachael Elliott, Yusuf Alwatani

**Affiliations:** 1 Rotherham Doncaster and South Humber NHS Foundation Trust, Doncaster, United Kingdom; 2 Essex Parternship University NHS Foundation Trust, Essex, United Kingdom; 3 Leeds and York Partnership NHS Foundation Trust, Leeds, United Kingdom

## Abstract

**Aims:**

Clinicians working in an Older People’s Mental Health (OPMH) inpatient unit noted that blood tests, particularly urea and electrolytes (U&E), were frequently completed when patients were commenced on psychotropic medications. An audit was undertaken to describe current practice and assess its alignment with relevant guidance. NICE and BNF guidelines do not recommend routine blood monitoring for memantine or pregabalin. RCPath advises renal function testing only if significant clinical changes indicate acute renal or electrolyte-related issues. Additionally, NHS England guidance advises all blood tests should have a documented rationale.

**Methods:**

Case notes of 30 OPMH inpatients who were initiated on memantine and/or pregabalin and had further blood tests taken after initial admission bloods between June 2023 and June 2024 were reviewed. The audit evaluated:

(1) Whether blood tests had a documented rationale

(2) Their temporal association with initiation or titration of these medications.

**Results:**

92 blood tests were taken for 30 patients. Rationale was documented for 62% of tests (n=37). Of those with rationale documented, 58% had a clinical indication e.g. suspected infection, general deterioration. Monitoring renal function due to psychotropic medication was documented as the rationale for 42% (n=24), all patients had previously normal kidney function. Among tests without a documented rationale, 25% (n=23) may have related to memantine or pregabalin initiation or titration. A temporal pattern was observed, with dose changes sometimes occurring shortly after U&E testing.

**Conclusion:**

A notable proportion of blood tests lacked a documented rationale and observed monitoring practices for memantine and/or pregabalin did not consistently align with current guidance. The observed temporal pattern may suggest an association, but this represents our own interpretation of the available documentation. Some tests may have been clinically justified but not recorded. While routine blood testing can support early detection of clinical issues, it also carries potential drawbacks, including patient distress and incidental findings that may prompt further investigations. We recommend multi disciplinary discussions to review the rationale for current monitoring practices, encourage adherence to guideline recommendations, and clear documentation of rationale for all blood tests.

